# The Molecular Mechanisms of Ferroptosis and Its Role in Blood-Brain Barrier Dysfunction

**DOI:** 10.3389/fncel.2022.889765

**Published:** 2022-05-19

**Authors:** Xiaoshu Chen, Xinru Pang, Abrey J. Yeo, Siwen Xie, Mengting Xiang, Bin Shi, Gongchang Yu, Chao Li

**Affiliations:** ^1^Shandong Academy of Occupational Health and Occupational Medicine, Shandong First Medical University and Shandong Academy of Medical Sciences, Jinan, China; ^2^University of Queensland Centre for Clinical Research, Brisbane, QLD, Australia; ^3^Neck-Shoulder and Lumbocrural Pain Hospital of Shandong First Medical University, Shandong First Medical University and Shandong Academy of Medical Sciences, Jinan, China

**Keywords:** ferroptosis, blood brain barrier, iron accumulation, lipid peroxidation, tight junctions

## Abstract

The blood-brain barrier (BBB) is a selective, semi-permeable layer of endothelial cells that protects the central nervous system from harmful substances circulating in blood. It is one of the important barriers of the nervous system. BBB dysfunction is an early pathophysiological change observed in nervous system diseases. There are few treatments for BBB dysfunction, so this motivates the review. Ferroptosis is a novel cell death mode caused by iron-mediated lipid peroxidation accumulation, which has recently attracted more attention due to its possible role in nervous system disorders. Studies have shown that lipid peroxidation and iron accumulation are related to the barrier dysfunction, especially the expression of tight junction proteins. Therefore, examination of the relationship between ferroptosis and BBB dysfunction may reveal new targets for the treatment of brain diseases.

## Introduction

**FIGURE 1:**
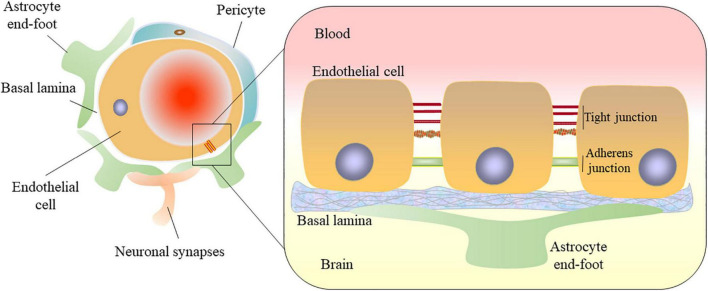
Structural diagram of the blood-brain barrier. The blood-brain barrier (BBB) consists of microvascular endothelial cells. Endothelial cells are connected by tight junctions and adherens junctions. The BBB surrounded by basal lamina, pericytes, and astrocytic perivascular end-feet in the neurovascular unit (NUV). Astrocytes provide the cellular link to the neurons. All of these come together to maintain the BBB structural and functional integrity.

Blood-brain barrier (BBB) is composed of specialized brain microvascular endothelial cells lining the vasculature of the central nervous system (CNS). The BBB surrounded by basal lamina, pericytes, and astrocytic perivascular end-feet and acting together with neurons and glial cells forms the neurovascular unit (NVU) which is crucial for the function of the brain (Zlokovic, 2011). Endothelial cells connected by tight junctions and adherens junctions form the tightly sealed wall of all cerebral vessels (Hawkins and Davis, 2005). Pericytes cover the vascular endothelium and regulate vascular stability, diameter, cerebral blood flow, and extracellular membrane protein secretion (Zheng et al., 2020). Astrocytes span around the vascular endothelium and pericytes *via* end-feet, and regulate BBB permeability (Abbott et al., 2006). All of these come together to maintain the BBB structural and functional integrity (Figure 1). From a physiological point of view, this barrier is generated not only by the morphological entity, but also by a variety of proteins and enzymes in these cells that work together as a controlled and selective transport and metabolic system, protecting the brain from dangerous polar chemicals in the bloodstream and to maintain a tightly regulated microenvironment for neuronal signaling (Hawkins and Davis, 2005). Adherens junctions (AJs) connect endothelial cells and tight junctions (TJs) limit the paracellular permeability of the BBB (Zlokovic, 2008). TJs are adhesions between endothelial cells that are composed of multiple transmembrane proteins, including claudin, occludin, junctional adhesion molecule, and zonula occludens-1 (ZO-1). They interact directly with extracellular components to connect two cell membranes together (Zlokovic, 2011; Tiwari et al., 2019). AJs are composed of vascular endothelial cadherin and connexin, which constitute the basic cell adhesion between endothelial cells, support the integrity of blood vessels and regulate tension, to limit the movement of solutes near and across cells. Vascular endothelial cadherin is the principal cadherin that forms AJs and mediates intercellular adhesion (Zenaro et al., 2017). Both TJs and AJs play key roles in the control of endothelial permeability (Yu et al., 2020). BBB breakdown and dysfunction leads to leakage of harmful blood components into the CNS, cellular infiltration, and aberrant transport and clearance of molecules. The integrity of the BBB remains vital for homeostasis and neural protection throughout life. The permeability of the BBB will change if the tight adhesion between endothelial cells is broken, affecting the central nervous system. In addition, BBB dysfunction is one of the early pathophysiological changes observed in neurodegenerative disorders and brain injury, such as stroke, traumatic brain injury (TBI), Alzheimer’s disease (AD) and Parkinson’s disease (PD) (Zlokovic, 2008; Yang et al., 2019; Cash and Theus, 2020). There is a growing body of evidence that ferroptosis plays a central role in several diseases, amongst those mentioned above (Magtanong and Dixon, 2018; Reichert et al., 2020).

Ferroptosis is a new type of cell death that was discovered in recent years and is usually accompanied by a large amount of iron accumulation and lipid peroxidation during the cell death process (Li et al., 2020). Morphologically, ferroptosis mainly manifests as a loss of plasma membrane integrity, cytoplasmic swelling (oncosis), swelling of cytoplasmic organelles and moderate chromatin condensation and obvious shrinkage of mitochondria with increased membrane density and reduction in or vanishing of mitochondrial cristae, which is a different process from other modes of cell death (Liang et al., 2019). Ferroptosis-inducing factors can directly or indirectly affect the imbalance of oxidative stress and antioxidant system through different ways, such as inhibiting glutathione synthesis, inactivating glutathione peroxidase 4 (GPX4), accumulating lipid reactive oxygen species (ROS), and finally leading to cell death (Cao and Dixon, 2016). Ferroptosis has been extensively reported to be involved in various neurological disorders. However, its specific role and mechanism in the BBB remain unclear. Understanding the regulatory mechanisms of ferroptosis in BBB will provide a new approach to prevent and treat neurological disorders. In this manuscript, we review the mechanism of ferroptosis and its role in BBB dysfunction and provide a new direction for uncovering novel therapeutic targets.

## The Mechanism of Ferroptosis

**FIGURE 2:**
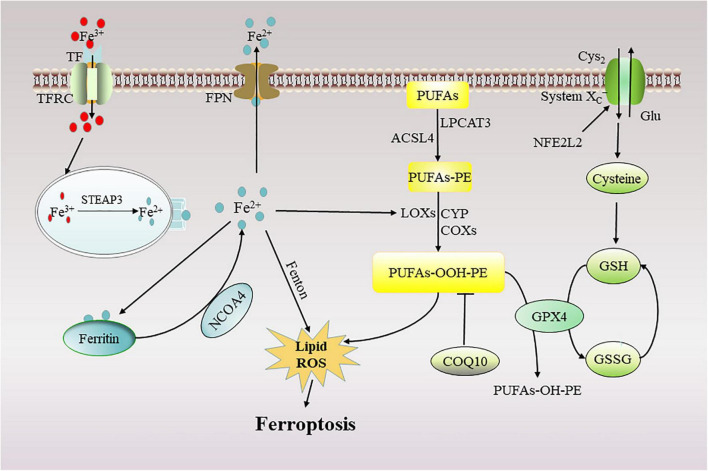
Mechanism of ferroptosis. Ferroptosis can occur in two major pathways: (1) through the accumulation of ferrous ions and (2) through the accumulation of lipid peroxides. TF, transferrin; TFRC, transferrin receptor; FPN, ferroportin; NFE2L2, the nuclear factor erythroid 2-like 2; STEAP3, 6-transmembrane epithelial antigen of the prostate 3; NCOA4, nuclear receptor co-activator 4; ROS, reactive oxygen species; GSH, glutathione; GPX4, glutathione peroxidase 4; PUFAs, polyunsaturated fatty acids; ACSL4, acyl-CoA synthetase long-chain family member 4; LPCAT3, lysophosphatidylcholine acyltransferase 3; CoQ10, Coenzyme Q10; Glu, glutamate; Cys_2_, Cystine; LOXs, lipoxygenase; CYP, cytochrome P450; COXs, Cyclooxygenases.

Ferroptosis is a novel mechanism of cell death caused by iron-mediated lipid peroxidation accumulation (Dixon et al., 2012). Regulating the balance between oxidative stress and the antioxidant system is an important molecular mechanism of ferroptosis (Kuang et al., 2020; Figure 2).

### Oxidative Damage in Ferroptosis

#### Iron Accumulation

High iron level may directly generate excessive ROS through the Fenton reaction, thereby increasing oxidative damage. In addition, iron may increase the activity of iron-containing enzymes such as lipoxygenase (LOXs), which are enzymes responsible for lipid peroxidation and oxygen homeostasis (Chen et al., 2020). Iron has two oxidation states: ferrous iron (Fe^2+^) or trivalent iron (Fe^3+^). The transferrin receptor (TFRC) on the cell membrane recognizes external Fe^3+^ binding to transferrin (TF) and imports it into the cytoplasm (Hou et al., 2016). Subsequently, iron reductase six transmembrane prostate epithelial antigen 3 (STEAP3) converts Fe^3+^ to Fe^2+^. The solute carrier family 11 divalent metal transporter 1 (SLC11A2/DMT1) transports Fe^2+^ from endocytosis into the cytoplasm, increasing intracellular iron content (Masaldan et al., 2019). In addition, nuclear receptor co-activator 4 (NCOA4)-mediated autophagic degradation of iron storage proteins [including ferritin light chain (FTL) and ferritin heavy chain 1 (FTH1)] can also increase the level of intercellular iron (Gao et al., 2015; Wang Y. et al., 2020). Excess iron can trigger the formation of highly destructive hydroxyl radicals through the Fenton reaction, and catalyze lipid peroxidation in cell membranes through LOXs, etc., thereby promoting ferroptosis (Wang et al., 2016).

#### Lipid Peroxidation

Lipid peroxidation is an important feature of ferroptosis, which mainly effects unsaturated fatty acids in the cell membrane. Lipid peroxidation refers to the process in which an oxygen or hydrogen peroxide molecule produces a hydroperoxy group, which is then inserted into a lipid molecule (Gaschler and Stockwell, 2017). Saturated fatty acids (no double bonds), monounsaturated fatty acids (MUFAs) with one double bond, and polyunsaturated fatty acids (PUFAs) with more than one double bond are the three forms of fatty acids. PUFAs, particularly arachidonic and adrenic acids, are particularly vulnerable to peroxidation during ferroptosis due to the presence of bis-allylic protons that are vulnerable to hydrogen atom abstraction (Yang et al., 2016), which can destroy the lipid bilayer and impair membrane function (Doll et al., 2017). Free PUFAs are esterified to membrane phospholipids, particularly arachidonic acid (AA) and adrenic acid (AdA). Membrane phospholipids are converted to arachidonic acid or adrenic acid coenzyme A (AA/AdA-CoA) and arachidonic acid or adrenic acid-phosphatidylethanolamine (AA/AdA-PE) by two enzymes, acyl-CoA synthetase long-chain family member 4 (ACSL4) and lysophosphatidylcholine acyltransferase 3 (LPCAT3). Lipid peroxides, a key aspect of ferroptosis, are formed in the final step: Through an enzymatic process involving LOXs and a non-enzymatic pathway known as Fenton-type chemistry, AA/AdA-PE is converted to lipid peroxides arachidonic acid or phospholipid hydroperoxides (AA/AdA-OOH-PE) inducing ferroptosis (Kagan et al., 2017).

### Antioxidant System in Ferroptosis

#### Glutathione Metabolism and System X_*C*_^–^

Cystine/glutamate-related amino acid metabolism is crucial in ferroptosis (Yang et al., 2014). Cystine is delivered to the cell and is subsequently reduced to cysteine, which forms glutathione (GSH) when combined with glutamic acid and glycine. The transmembrane cystine-glutamate antiporter system X_*C*_^–^ is made up of two chains: solute carrier family 7 member 11 (SLC7A11) and solute carrier family 3 member 2 (SLC3A2). System X_*C*_^–^ transports cystine into the cell and glutamate or cysteine out of the cell in a 1:1 exchange ratio (Stockwell et al., 2017). GSH, a tripeptide antioxidant, is then used as a cofactor for glutathion peroxidase 4 (GPX4), which reduces oxidative stress, maintains the intracellular redox balance and inhibits ferroptosis (Xu et al., 2021). The synthesis of GSH depends on the availability of cysteine (generated from its precursor cystine), the level of sulfur amino acid precursors and the activity of glutamate-cysteine ligase (GCL) (Kuang et al., 2020). The inhibition of system X_*C*_^–^ (using erastin, sorafenib, and sulfasalazine) or GCL (using buthionine sulfoximine) triggers ferroptosis in various cells (Jiang et al., 2015). The expression or activity of System X_*C*_^–^ is affected by epigenetics, transcription, and posttranscriptional and posttranslational regulators, such as a tumor protein p53 (p53), the nuclear factor, erythroid 2-like 2 (NFE2L2), BRCA1-associated protein 1 (BAP1), or mucin 1 cell surface-associated (MUC1), leading to complex feedback mechanisms to control GSH levels in ferroptosis (Hayano et al., 2016; Chen et al., 2017; Song et al., 2018; Zhang Y. et al., 2018).

#### Glutathione Peroxidase 4

Glutathione peroxidase 4 functions as a phospholipid hydroperoxidase to reduce phospholipid hydroperoxide production to the corresponding phospholipid alcohol (Yang et al., 2014; Tang et al., 2021). Selenium (Se) and GSH regulate the expression and activity of GPX4. The function of selenium as an essential trace element is dependent on a single functional group, the selenol group. Active selenol is oxidized by peroxide to selenic acid, which is then reduced by GSH to intermediate selenide disulfide in the GPX4 catalytic cycle. The second GSH activates GPX4, causing glutathione disulfide (GSSG) to be released. Selenolate-based catalysis of the essential mammalian selenoprotein GPX4 is indispensable for normal embryogenesis (Ingold et al., 2018). Se drives the transcriptional activators *TFAP2c* and *Sp1* to upregulate GPX4, against ferroptosis in neurons resulting in neuroprotection. The study found that a single dose of Se delivered into the brain drives antioxidant GPX4 expression, protects neurons, and improves behavior in a hemorrhagic stroke model (Alim et al., 2019). GPX4 inhibitors are also known as classic ferroptosis activators, although their activities and effects differ, some compounds also cause GPX4 protein degradation (Shimada et al., 2016; Muller et al., 2017; Zhu et al., 2017; Gaschler et al., 2018).

#### Non-GPX4 Pathways

Several non-GPX4 pathways, including the coenzyme Q10 (CoQ10) system and endosomal sorting complexes required for transport (ESCRT)-III pathway membrane repair systems, also play an important role in protecting against oxidative damage during ferroptosis (Bersuker et al., 2019; Doll et al., 2019; Dai et al., 2020a; Kraft et al., 2020). CoQ10 is a strong antioxidant that promotes the mitochondrial respiratory chain while also neutralizing free radicals in various membrane structures (Teran et al., 2018). CoQ10 also inhibits apoptosis (Chen et al., 2011), and ferroptosis (Shimada et al., 2016). In particular, apoptosis-inducing factor mitochondrial-related 2 (AIFM2), a conventional regulator of apoptosis in the mitochondria (Wu et al., 2002), may block ferroptosis in a GSH-independent manner by mediating the synthesis of CoQ10. *N*-myristoylation of AIFM2 is required for this step, which results in AIFM2 translocation to the cell membrane (Bersuker et al., 2019; Doll et al., 2019). The depletion of CoQ10 biosynthesis enzyme [coenzyme Q2, polyprenyltransferase (CoQ2)] may reverse the anti-ferroptotic activity of AIFM2 (Bersuker et al., 2019; Doll et al., 2019). Of note, increased AIFM2 levels in the cell membrane may also prevent ferroptosis by activating ESCRT-III membrane repair pathways mediated by charged multivesicular body protein 5 (CHMP5) and CHMP6 that are not dependent on CoQ10 (Dai et al., 2020b). Tetrahydrobiopterin (BH4) production and recycling is a dynamic process, with GTP cyclohydrolase-1 (GCH1) serving as the rate-limiting enzyme. GCH1-mediated BH4 synthesis inhibits lipid peroxidation, preventing ferroptosis and demonstrating that BH4 possesses antioxidant function during cell death (Kraft et al., 2020).

## Ferroptosis in Blood-Brain Barrier Degradation

### Lipid Peroxidation in Blood-Brain Barrier

Lipids are important components of cell membranes and are abundant in the central nervous system. Lipid peroxidation changes the fluidity and permeability of cell membrane, and finally leads to the change of cell structure and function. The central nervous system is vulnerable to overwhelming lipid peroxidation because of its special structure (Anthonymuthu et al., 2016). There are three well-defined classes of lipid oxidation enzymes: cyclooxygenases (COXs), cytochrome p450 (CYP), and LOXs (Chauhan et al., 2019), which are involved in the process of iron-dependent lipid peroxidation and can catalyze the conversion of PUFAs to lipid hydroperoxides. In astrocytes CYP isoforms are expressed at a very high level, which form a metabolic barrier regulating drugs’ influx, modulate blood-flow regulation, and act as signaling enzymes in inflammation (Meyer et al., 2007). CYP suppression ameliorates cerebral ischemia reperfusion (I/R) injury and protects BBB integrity by reducing both oxidative stress and inflammation (Yu et al., 2021). COXs are abundantly expressed in neurons and microglia across various animal species (Khan et al., 2022). There is considerable evidence indicating that COXs are important in BBB permeability changes. Studies have found that lipopolysaccharide (LPS) induces the destruction of BBB through a COX-dependent pathway (Banks et al., 2015; Cheng et al., 2018). In addition, *in vivo* and *in vitro* experimental showed that tumor necrosis factor-α (TNF-α)-mediated BBB breakdown is linked to COXs up-regulation the expression and activity of matrix metalloproteinases (MMPs) (Mark et al., 2001; Candelario-Jalil et al., 2007). It has already been shown in human aortic endothelial cells that lipid peroxidation activates MMP-2/9, which contributes to the development of endothelial dysfunction (Sugimoto et al., 2009). The same was found in another study where increased lipid peroxidation and low-density lipoprotein oxidation were observed in the brains of hyperlipidemic mice and this was associated with increased activation of calpain-1/2 and MMP-2/9, and the downregulation of the TJs protein occludin in cerebral microvessels (ElAli et al., 2011). A recent study employed brain microvascular endothelial cells; to investigate the effects of p53 on the lipid oxidation of the BBB. This suggests that the expression levels of p53 regulate the oxidative stress and lipid peroxidation in regulating the permeability/integrity of the BBB cells, and that p53 supports BBB integrity, at least in part, by reducing lipid peroxidation (Akhter et al., 2019).

The intake of high doses of polyunsaturated fatty acids can promote lipid peroxidation and the subsequent propagation of oxygen radicals. Previous studies have found that the mechanism of ROS-induced barrier dysfunction in endothelium is by destroying TJs the same as that in epithelial cells (Rao, 2008). A study was to show the effect of dietary enrichment with docosahexaenoic acid (DHA) on lipid peroxidation and TJs structure and permeability in Caco-2 cell cultures. They found that incubation with 100 mM DHA increased lipid peroxidation and paracellular permeability, in parallel with a redistribution of the TJs proteins occludin and ZO-1 (Roig-Perez et al., 2004). Another study yielded the same results, it showed that the disruption of epithelial barrier function by DHA is partly mediated by the formation of eicosanoids (Roig-Perez et al., 2010). The products of lipid peroxidation include the initial lipid hydroperoxides and subsequent reactive aldehydes malondialdehyde (MDA) and 4-hydroxynonenal (4-HNE), which increase during ferroptosis (Kagan et al., 2017; Wenzel et al., 2017). A study also showed that a systemic treatment with 4-HNE suppressed colonic expressions of TJs protein occludin, impaired intestinal barrier function (Wang Y. et al., 2019). It has been found the increase in the content of MDA, reduced the viability of pig iliac endothelium cells, and the barrier functions were destroyed, along with the down-regulations on claudin-1, occluding, and ZO-1 and the increase of paracellular permeability (Yuan et al., 2019). Phospholipid transfer protein (PLTP) is a protein that regulates lipid metabolism and is involved in oxidative stress. In PLTP-deficient animals, cerebral oxidative stress increased, with higher levels of ROS and the lipid peroxidation marker 4-HNE, as well as lower superoxide dismutase (SOD) activity. In addition, PLTP knockout mice had enhanced BBB permeability and reduced expression of the tight junction proteins occludin, ZO-1, and claudin-5 in brain arteries. Vitamin E supplementation also improved BBB integrity and TJs protein expression by lowering cerebrovascular oxidative stress (Zhou et al., 2014).

**FIGURE 3:**
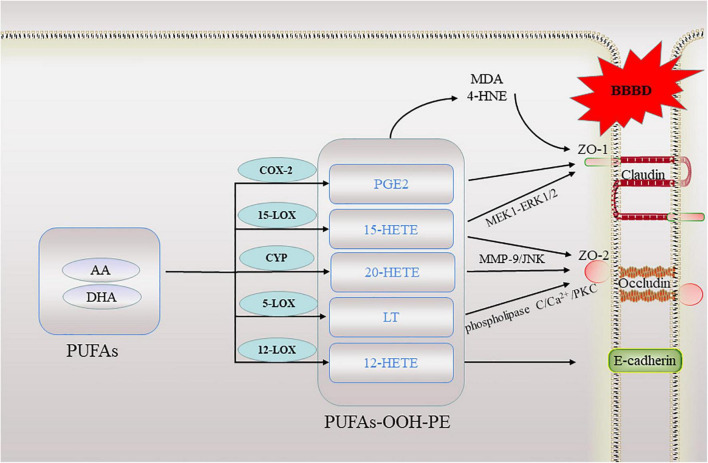
A schematic of lipid peroxidation occurring in the blood brain barrier. When ferroptosis occurs, lipid peroxidation directly or indirectly involved in this process destroy the tight junctions between cells in the blood-brain barrier. BBBD, blood-brain barrier dysfunction; AA, arachidonic acid; DHA, docosahexaenoic acid; ZO, zonula occludens; 20-HETE, 20-hydroxyeicosatetraenoic acid; 15-HETE, 15-hydroxyeicosatetraenoic acid; 12-HETE, 12-hydroxyeicosatetraenoic acid; LT, Leukotrienes; PGE2, prostaglandin E2; PKC, protein kinase C; MMP, matrix metalloproteinases; MDA, malondialdehyde; 4-HNE, 4-hydroxynonenal.

In addition to the above mentioned, some lipid metabolites of AA are also known to contribute to high barrier permeability. 15-hydroxyeicosatetraenoic acid (15-HETE), the major 15-LOXs metabolite of AA, by stimulating ZO-2 tyrosine phosphorylation and its dissociation from claudin-1/5, induces endothelial TJs disruption and its barrier dysfunction (Chattopadhyay et al., 2015). Further research found that 15-HETE enhances ZO-1 phosphorylation *via* PKCε-mediated MEK1-ERK1/2 activation, causing ZO-1 dissociation from occludin, disrupting endothelial TJs and its barrier function, and promoting monocyte transmigration. In line with these observations, in WT mice high fat diet feeding induced 12/15-LOXs expression in the endothelium and caused disruption of its TJs and barrier function (Chattopadhyay et al., 2019). 20-hydroxyeicosatetraenoic acid (20-HETE) is a metabolite of arachidonic acid, a study suggested that 20-HETE may aggravate BBB disruption, *via* enhancing the expression of MMP-9 and TJs proteins. Furthermore, oxidative stress and the JNK signaling pathway may be involved in BBB dysregulation (Lu et al., 2018). AA mediates permeability of human brain microvascular endothelial cells through prostaglandin E2 (PGE2) (Dalvi et al., 2015). In addition, direct treatment of brain microvessels with PGE2 resulted in decreased expression of both occludin and ZO-1 (Pu et al., 2007). Leukotrienes (LT) also are inflammatory mediators derived from arachidonic acid. A study showed that infusion of LTC4 into rat carotid artery is used to selectively open the BBB in ischemic tissue (Baba et al., 1991). The eicosanoids produced by the 5-LOX pathway, LTB4 and D4, and 5-hydroxyeicosatetraenoic acid activate the phospholipase C/Ca (2+)/protein kinase C pathway, resulting in increased paracellular permeability and the redistribution of occludin, which are involved in the destruction of barrier function (Rodriguez-Lagunas et al., 2013). The lipoxygenases metabolite, 12-hydroxyeicosatetraenoic acid (12-HETE), can impair vascular endothelial permeability by altering AJs phosphorylation levels (Wang X. et al., 2019). These evidences have shown that lipid peroxidation destroys TJs and affects barrier function (Figure 3).

### Iron Dysmetabolism in Blood-Brain Barrier

Through its participation in many cellular activities such as mitochondrial respiration, myelin synthesis, and neurotransmitter synthesis and metabolism, iron in the brain plays a critical role in maintaining normal physiological function. Because the endothelial cells of the BBB are a regulatory site for brain iron uptake, proper structure and function of the BBB is vital for brain iron homeostasis. The pericytes and astrocytes of the neurovascular unit surround the abluminal side of brain capillary endothelial cells, providing extra structural and functional support. Brain microvascular endothelial cells lack fenestrations and possess TJs, forcing most molecules to be trafficked transcellularly *via* receptor mediated transcytosis, adsorptive trans cytosis, or transport proteins (Hawkins and Davis, 2005). The transcellular trafficking of iron from the blood into the brain interstitium depends on iron uptake proteins in the apical membrane of brain microvascular capillary endothelial cells and efflux proteins at the basolateral, abluminal membrane. The study results imply that BBB endothelial cells can also regulate the two possible iron transport pathways (transferrin-bound iron and non-transferrin-bound iron) by controlling receptor expression, internalization of transferrin receptor complexes, and acidification inside cell endosomes (Khan et al., 2018). Microvascular cell iron availability and efflux have been found to be tightly modulated by diffusible ceruloplasmin and hepcidin released from astrocytes surrounding the BBB (McCarthy and Kosman, 2014). Iron is essential for supporting brain function, yet over-accumulation is cytotoxic. The management of iron flux by the BBB provides the first line of defense against the over-accumulation of iron in normal physiology and in pathological conditions.

However, the failure of uptake, transport, storage, and utilization of intracellular iron can lead to excess intracellular free Fe^2+^ deposition. This over-accumulation of iron is common to several neurological disorders, these include Alzheimer’s disease, Parkinson’s disease and other disorders presenting with neurodegeneration and associated brain iron accumulation (Reichert et al., 2020). There is a lot of evidence that various neurological diseases can lead to BBB dysfunction, but the mechanism of this disease is not clear. Recently, a study has shown that an iron chelator rescued the brain microvascular endothelial cells from both toxicity and functional damage, suggesting that an increase of iron in the brain microvascular endothelial cells was responsible for the BBB breakdown (Rand et al., 2021). In addition, studies performed in rats determined whether transient forebrain ischemia (TFI) causes the increase in BBB permeability and neuronal death. It is possible that iron overload and iron-mediated free radical production cause the loss of TJs proteins and degeneration of endothelial cells, and that targeting iron mediated oxidative stress provides an extended therapeutic time window against an ischemic event (Park et al., 2011; Won et al., 2011). Excess iron accumulation induced MMPs expression (Woo et al., 2020). Studies have suggested that MMPs degrade the vascular basement membrane components, resulting in disruption of the BBB (Jin et al., 2019), and that excess iron accumulation will initiate the Fenton reaction to generate ROS (Xu et al., 2020). ROS subsequently modifies and interferes with proteins, lipids, and DNA, which can induce cell death. Experimental evidence and clinical data suggest that ROS disrupts the BBB by degrading the TJs (Sandoval and Witt, 2008). Furthermore, it was demonstrated that iron can alter TJs permeability of Caco-2 cells in monolayer cultures (Ferruzza et al., 2003). TJs injuries were observed by fluorescent staining in iron-overloaded wild-type mice that did not have leaky gut or endotoxemia (Visitchanakun et al., 2021). Another study reported that hemin-induced BBB damage and neuropathy showed intracellular Fe^2+^ accumulation in endothelial cells and caused pericytes-induced endothelial barrier dysfunction. Hemin was also shown to disrupt the VE-cadherin integrity (Imai et al., 2019). Hence, there is accumulating evidence that iron regulates the integrity of TJs and can therefore influence BBB function.

### The Role of Reactive Oxygen Species in Blood-Brain Barrier

Reactive oxygen species is a by-product of cell metabolism. During normal physiological metabolism, ROS regulates various cellular function and participates in cell signaling. ROS is also critical in ferroptosis. It is generated when molecular oxygen is partially reduced to superoxide, hydrogen peroxide, lipid peroxides, or the hydroxyl and peroxyl radicals (Gaschler and Stockwell, 2017). The primary biological sources of ROS are mitochondrial metabolism and the cell membrane enzyme nicotinamide adenine dinucleotide phosphate oxidase (NOX). NOX1, cytochrome B-245 beta chain (CYBB/NOX2), NOX3, NOX4, NOX5, dual oxidase 1 (DUOX1), and DUOX2 are members of the NOX family. They’re part of a membrane-bound enzyme complex that can transport electrons across the plasma membrane to create superoxide and other downstream ROS with the help of other proteins. ROS generation mediated by NOX1, CYBB/NOX2, and NOX4 accelerates lipid peroxidation in ferroptosis (Poursaitidis et al., 2017; Xie et al., 2017; Yang W. H. et al., 2020). As mentioned above, iron participates in ROS accumulation and this can be through three pathways. Firstly, intracellular free Fe^2+^ causes the Fenton reaction, which is an inorganic chemical reaction that involves peroxides and Fe^2+^ to produce soluble hydroxyl or lipid alkoxy radicals. Second, ROS is produced *via* lipid autoxidation, a classic autocatalytic free radical chain reaction that produces lipid hydroperoxides in the presence of iron. Finally, iron has a role in the catalytic subunit of LOXs, which oxidize PUFAs to produce lipid peroxides (Dixon and Stockwell, 2014). A study showed that iron-mediated ROS leads to the down-regulation of TJs protein expression and endothelial cell dysfunction, thereby promoting increase of in the BBB permeability (Won et al., 2011).

Reactive oxygen species activate enzymes and signal cascades that involve lipids and chromatin, and it also changes the expression, distribution and phosphorylation of TJs proteins to compromise barrier function. In fact, experiments have shown that ROS can alter BBB permeability by influencing ZO protein distribution. For instance, exposure to H_2_O_2_ led to a redistribution of ZO-1 from the TJs to the cytosol, resulting in a decrease in transepithelial electrical resistance (TEER) and an increase in BBB permeability (Lee et al., 2004). In addition, alcohol-induced oxidative stress was found to increase serine phosphorylation on claudin-5 and occludin and serine/threonine phosphorylation on ZO-1 (Carrino et al., 2021). Increased serine phosphorylation has been shown to decrease BBB resistance (Ishizaki et al., 2003). ROS can change the vascular tone and therefore influence cerebral blood flow. These vascular effects also include increasing platelet aggregability and endothelial cell permeability, altering reactivity to vasodilators, and leading to the formation of focal lesions in endothelial cell membranes (Correale and Villa, 2007). Active oxygen can also interact with MMPs that finally result in BBB dysfunction (Lin et al., 2015). It has been found that NOX-mediated oxidative stress promotes the permeability of BBB by increasing the activity of MMPs, and NOX2 is an important source of ROS in astrocyte and vascular endothelial cells. In addition, the gene deletion of catalytic subunit gp_91_^phox^ of NOX2 weakened the BBB damage induced by ischemic stroke in gp_91_^phox^-deficient mice to a great extent compared with wild-type mice (Kahles et al., 2007). The BBB protection seen in ischemic gp_91_^phox^ deficient mice is likely the result of less induction of MMP-9 and reduced loss of occludin, a critical TJs of the BBB (Liu et al., 2011). Increasing evidence suggest that ROS can induce TJs rearrangement or degradation of endothelial cells, leading to increased paracellular permeability and disrupt the BBB (Van der Goes et al., 2001; Rao et al., 2002; Witt et al., 2003; Sandoval and Witt, 2008). The importance of astroglial cells for the defense of the brain against ROS and especially the function of astroglial glutathione metabolism has become evident at least for cell culture models. *In vivo*, a compromised astroglial glutathione system may contribute to a lower defense capacity of the brain against ROS and subsequently to increased susceptibility to ROS of astrocytes themselves and of neighboring cells (Dringen, 2000) and compromises barrier integrity.

### The Role of Glutathione Peroxidase 4 in Blood-Brain Barrier

Glutathione peroxidase 4, a GSH-dependent enzyme downstream of the system, assists to maintain membrane fluidity by reducing lipid peroxides. To protect cells from deadly lipid ROS deposition, GPX4 converts GSH into oxidized glutathione (GSSG) and reduces lipid peroxides (L-OOH) to the respective alcohols (L-OH) (Stockwell et al., 2020). Studies have found that mice with gene knockout or pharmacological inactivation of GPX4 have reduced numbers of surviving neurons, dysfunction of the BBB, and aggravated brain edema. Ferrostatin-1 treatment can significantly increase the protein level of GPX4 in brain tissue after intracerebral hemorrhage and reduce the BBB damage caused by intracerebral hemorrhage (Zhang Z. et al., 2018). In another study, GPX4 knockout mice showed significant cognitive impairment and degeneration of hippocampal neurons when challenged with the water maze test. Treatment with a small-molecule ferroptosis inhibitor ameliorated neurodegeneration in those mice (Hambright et al., 2017), and induced the upregulation of GPX4 expression and inhibition of neuronal ferroptosis by reducing albumin extravasation and BBB injury (Peng et al., 2021). At present, GPX4 has become a key regulator or target in erastin-induced ferroptosis. GPX4 has been considered to be involved in eicosanoid synthesis by controlling COXs and LOXs activities. A study found that GPX4 effectively prevents oxidative stress–induced cell death by specifically controlling 12/15-LOXs (Schneider et al., 2010). Additionally, COXs and LOXs signaling pathways induce oxidative stress to break the integrity of BBB and increase its permeability by producing ROS. Another study found that selenium enhances GPX4 expression and total GPX activity, while knockdown of GPX4 by small interfering RNA increased vascular endothelial growth factor (VEGF) and Interleukin-8 expression (Rohr-Udilova et al., 2012). VEGF has been characterized as an inducer of vascular leakage in response to hypoxia (Sandoval and Witt, 2008). It has also been reported that VEGF alters the expression and distribution of TJs proteins, leading to BBB hyperpermeability in hypoxia and autoimmune encephalomyelitis (Argaw et al., 2009), indicating that VEGF plays a major role in BBB disruption (Zhang et al., 2000). In conclusion, these evidences suggest that GPX4 can indirectly regulate the integrity of BBB.

### Others

During ferroptosis, NFE2L2 acts as a master transcription factor, coordinating the activation of a number of cytoprotective genes involved in iron metabolism, oxidative defense, and redox signaling. Nuclear factor erythroid 2-related factor 2 (Nrf2) is a transcription factor encoded by NFE2L2, which is considered a master regulator of the antioxidant response. Some studies have confirmed that GPX4 and cystine/glutamate transporter system are downstream targets of Nrf2 (Dodson et al., 2019). Early studies have demonstrated the protective role of Nrf2 against TJs degradation and attenuated BBB function after various types of brain injury (Jadhav et al., 2007; Kunze et al., 2015; Zhang et al., 2017). In several of these reports, Nrf2 has been demonstrated to upregulate the TJs proteins expression and maintain the BBB integrity (Alfieri et al., 2013; Algattas and Huang, 2013).

Coenzyme Q10, as a scavenger of superoxide dismutase in mitochondria, can stimulate mitosis, reduce lipid peroxidation in mitochondria, activate uncoupling protein, promote mitochondrial biosynthesis, and positively regulate the plasma membrane redox system (Morris et al., 2021). Studies have found that the use of CoQ10 can reduce tumor necrosis factor-α (TNF-α), and downregulate MMP-2,9 (Ghasemloo et al., 2021). The increase in levels of MMP, especially 2 and 9, can lead to the damage of various proteins in TJs and BBB basement membrane, contributing to the destruction of the integrity of BBB. It has already been observed *in vitro* that treatment with CoQ10 prior to cell exposure to ultraviolet could increase astrocytes viability (Jing et al., 2015). In addition, CoQ10 can effectively reduce the production of ROS, thereby reducing ischemic neuronal damage (Eftekhari et al., 2016). In addition, pretreatment with CoQ10 appears to ameliorate the diabetic hyperglycemia aggravated I/R brain damage in the middle cerebral artery occlusion (MCAO) of rats by maintaining the balance between mitochondrial fission and fusion (Lu et al., 2017). Therefore, CoQ10 may play a positive role in improving BBB function and reducing tissue damage.

## Targeting Ferroptosis to Restore Blood-Brain Barrier Function

### Antioxidant Therapy

Blood-brain barrier injury significantly accelerates the progression of brain diseases and antioxidant therapy may prevent BBB injury. Pharmacological inhibition of COXs significantly reduced MMP-3 and MMP-9 protein expression and enzymatic activity in an *in vivo* and *in vitro* model of brain injury can explain the efficacy of COXs inhibitors in protecting against BBB disruption following cerebral ischemia, bacterial meningitis, and against edema associated with brain tumors (Mark et al., 2001; Candelario-Jalil et al., 2007; Yang C. et al., 2020). Studies have proposed treatment with melatonin and edaravone, which improve the permeability of BBB in rat models of subarachnoid hemorrhage. Melatonin and edaravone act as free radical scavengers. thereby reducing ROS levels and increasing the expression of antioxidant enzymes to facilitate its neuroprotective role (Ayer et al., 2008; Nakamura et al., 2008). The recent study found that in both *vivo and vitro* experiments, melatonin elevated TJs expression after TBI, thereby protecting the BBB. In addition, melatonin alleviated long-term sleep disorders and improved neurological function in TBI mice (Wu et al., 2022). Thus, these findings suggested that melatonin might potentially protect the injured brain by attenuating ferroptosis. Pretreatment with 3-n-Butylphthalide can significantly reduce BBB injury and brain edema in the model of middle cerebral artery occlusion, and its beneficial effect is related to reducing ROS, malondialdehyde and increasing SOD activity (Yan et al., 2017). Resistin is a cysteine rich peptide produced mainly by human macrophages (Jung et al., 2006). In a study employing mouse models, resistin was shown to exert beneficial effects on infarct size, BBB permeability and neurological function in a dose-dependent manner by inhibiting cellular oxidative stress (Behrouzifar et al., 2018). Several studies revealed that blockade of GSH production and secretion by siRNA-mediated knockdown of γ-glutamylcysteine ligase significantly compromised endothelial cell barrier integrity. Using different GSH donors, showed that exogenous GSH supplementation improves barrier function by maintaining TJs organization and preventing injury-induced TJs phosphorylation (Huang et al., 2020). *N*-acetylcysteine (NAC) is a cysteine donor and the cysteine is an amino acid required for glutathione synthesis. NAC plays a role in the scavenging of free radicals and enhancing the synthesis of GSH. NAC was shown to significantly increase GSH and diminish levels of malondialdehyde (byproduct of lipid peroxidation) in an immortalized endothelial cell line established from rat brain capillaries in a model of oxidative stress induced BBB disruption (Vorbrodt and Dobrogowska, 2004). NAC amide protects against oxidative stress in human brain microvascular endothelial cells and significantly protected the integrity of our BBB model, as shown by permeability and in TEER studies (Zhang et al., 2009). Hence, GSH treatment may be an effective way to combat endothelial cell dysfunction, promote BBB function and maintain vascular health.

### Iron Chelator

Iron accumulation plays an important role in ferroptosis, suggesting that iron chelation therapy may be feasible in the treatment of BBB disorders. Iron chelators are ferroptosis inhibitors that target free iron in labile iron pools and prevent iron from supplying electrons to oxygen to form ROS. Deferoxamine (DFX) is a metal chelating agent which can eliminate iron overload. A study reported the protective effect of DFX on mice with microbleeds, and found that DFX can improve the permeability of the BBB, iron deposition, microglia activation, and dendritic cell damage (He et al., 2016). In addition, studies have shown that DFX therapy can prevent vascular degenerative changes and microglial activation in diabetic rats, while improving BBB permeability (Abdul et al., 2021). In rat and mouse models, MCAO resulted in elevated iron levels in the lesioned hemisphere that correlated with a reduction in iron export. DFX preconditioning protects against cerebral ischemia significantly improving neurologic deficit by including chelation of the small fraction of unbound iron (the labile iron pool) responsible for catalyzing the production of reactive oxygen species and expressions of hypoxia-inducible factor 1 (Li et al., 2008; Hanson et al., 2009; Tuo et al., 2017). Iron chelation can reduce brain edema, damage in the BBB microstructure and neurological deficits and reverse cognitive impairment by protecting the integrity of BBB in hippocampus (Li et al., 2017). Recently, it was shown in another model of BBB injury induced by organophosphorus, that the iron chelator Desferal significantly decreased mitochondrial ROS formation and apoptosis subsequent to barrier insult, while also rescuing barrier integrity by inhibiting the labile iron pool increase, inducing hypoxia induced factor 2α expression and preventing the degradation of Ve-cadherin specifically on the endothelial cell surface (Rand et al., 2021). Evidence indicates that an altered BBB contributes to iron accumulation, particularly in substantia nigra and striatum, and neuroinflammation in PD models. Iron chelator treatment attenuated iron accumulation and suppressed neuroinflammation, which further decreased the BBB impairment and the damage of dopaminergic neurons (Olmedo-Diaz et al., 2017; Tai et al., 2020; Han et al., 2021; Riederer et al., 2021; Zhao et al., 2021). And in disease animal models of AD, accumulation of iron can not only prompt the accumulation and aggregation of the Aβ and tau protein, but also induces ROS production in the brain of AD. Ferroptosis inhibitors, such as ferrostatins-1 and liproxstatins-1, have been shown to protect neurons and recover cognitive function (Yamamoto et al., 2002; Bao et al., 2021). In conclusion, the intervention of ferroptosis related targets have achieved good effects *in vivo* and *in vitro* experimental models, which opens a new window for the study of neurological disease caused by BBB damage.

## Conclusion

The properties of the BBB are critical for proper neural function, and dysfunction of the BBB can lead to key pathologies in many neurological disorders. Nevertheless, whether ferroptosis plays a role in BBB dysfunction remains unclear. In this review, we explored the relationship between ferroptosis and BBB disorders. Ferroptosis is a new pathophysiological pathway discovered in recent years, which also provides a different perspective for the insight of nervous system diseases. Ferroptosis has been implicated in various neurodegenerative diseases however the exact role between ferroptosis and the BBB remains unclear. In addition, several questions remain unanswered: (A) The exact mechanism and other pathways of ferroptosis in the BBB, (B) the key targets of ferroptosis in the BBB, (C) the relationship between other forms of cell death, such as apoptosis, necrotic death, and ferroptosis, in the BBB disorders, and (D) whether drugs for ferroptosis can play an important role in the clinical treatment of BBB disorders. Although there is still a lot of investigation to be done before the results can be translated into clinical treatment, we believe that ferroptosis is a significant kind of cell death in brain diseases, and that further research into the phenomenon will open up new avenues for the diagnosis and treatment of nervous system disorders.

## Author Contributions

CL and GY designed and directed the project. XC wrote the manuscript. XC, XP, AY, SX, and MX collected materials. CL, GY, XC, and BS participated in the revision of the manuscript. All authors discussed the results and contributed to the final manuscript.

## Conflict of Interest

The authors declare that the research was conducted in the absence of any commercial or financial relationships that could be construed as a potential conflict of interest.

## Publisher’s Note

All claims expressed in this article are solely those of the authors and do not necessarily represent those of their affiliated organizations, or those of the publisher, the editors and the reviewers. Any product that may be evaluated in this article, or claim that may be made by its manufacturer, is not guaranteed or endorsed by the publisher.
